# The Relationship Between Gender Self-Stereotyping and Life Satisfaction: The Mediation Role of Relational Self-Esteem and Personal Self-Esteem

**DOI:** 10.3389/fpsyg.2021.769459

**Published:** 2022-01-07

**Authors:** Junnan Li, Yanfen Liu, Jingjing Song

**Affiliations:** ^1^Institution of Psychology, China University of Geosciences, Wuhan, China; ^2^Research Central for Psychology and Health Sciences, China University of Geosciences, Wuhan, China

**Keywords:** self-stereotyping, gender stereotype, relational self-esteem, life satisfaction, personal self-esteem

## Abstract

Individuals voluntarily internalize gender stereotypes and present personality characteristics and behaviors that conform to gender role requirements. The aim of the current study was to explore the reasons people internalize gender stereotypes. We conducted surveys with 317 college students in China to examine the relationship between gender self-stereotyping and life satisfaction. We also analyzed the mediating roles of relational self-esteem (RSE) and personal self-esteem (PSE) and the moderation role of gender. The results of path analysis showed that gender self-stereotyping directly affected life satisfaction and indirectly affected life satisfaction through RSE and PSE in a serial pattern; however, the serial mediation model was only significant in the male sample. Higher gender self-stereotyping was associated with male participants’ higher level of RSE and PSE and further correlated with higher life satisfaction. This study addressed the questions: “What are the benefits of gender self-stereotyping?” and “What are the major barriers to counter-stereotyping?” The results enrich our understanding of these issues, especially relative to the collectivist culture in China, and may be used to create more effective interventions to help people break through the stereotypes.

## Introduction

Many films and television shows in China portray grandmother figure who favor boy grandchild over girl grandchild. Although the grandmothers had themselves been victims of the traditional gender idea of “preferring boys to girls,” they eventually came to support that notion. Why is the influence of traditional gender ideas so profound? Why do people spontaneously fit into social roles, even when the social role is that of a socially disadvantaged group with low social status and low power (such as the role designated for women in some cultures)? One reason may be that meeting the requirements of social roles and self-stereotyping can induce a series of positive effects, such as improving one’s sense of belonging, improving interpersonal relationships, reducing the perception of discrimination, and improving life satisfaction, whereas violating stereotypical expectations would lead to negative consequences, such as more social pressure and poorer interpersonal relationships (Rudman and Glick, 1999; Brescoll et al., 2010; Hornung et al., 2019; Song and Liu, 2021). Empirical studies have shown that self-stereotyping is positively associated with well-being (Latrofa et al., 2009; Giamo et al., 2012), and women who violate their gender role requirements and appear to have more agency are discriminated against and judged for being “less feminine” (Rudman and Glick, 2001; Brescoll et al., 2010).

The current study focused on the relationship between gender self-stereotyping and life satisfaction and its’ mechanisms. The discussion on this issue has important theoretical and practical significance. The empirical research results can help us expand the self-categorization theory and social identity theory, gain a better understanding of why people voluntarily engage in gender self-stereotyping, and clarify the barriers preventing people from violating gender roles. Moreover, the results could be useful for improving or creating intervention methods to help free people from the constraints of gender stereotypes and further pursue self-realization.

## Background

### The Relationship Between Self-Stereotyping and Life Satisfaction

Self-stereotyping refers to a process by which people who belong to a stigmatized social group tend to describe themselves as having more stereotypical ingroup personality traits than non-stereotypical traits (Latrofa et al., 2009). In a broader definition, it is a process in which individuals integrate the stereotypes of the ingroup into their self-concept; this occurs for both those in the stigmatized social group and those in the advantaged social group (Becca, 1996, 2003; Biernat et al., 1996; Chiu et al., 2016). Self-categorization theory (Turner et al., 1987) holds that group members assimilating their attitudes and behaviors to the characteristic of the ingroup leads to attitude polarization between the two groups in contention (Han and Federico, 2018; Mason and Wronski, 2018). Self-classification actuates a group identity and activates group stereotypes (Turner et al., 1987). Moreover, stereotypes act as gender identity norms that affect a person’s view of themselves, thus leading individuals to present corresponding personality traits and behavioral responses according to the norms of the inner group (Smith et al., 2021). For example, due to the influence of gender roles and gender stereotypes, many women have long hair, are family oriented, have feminine personality traits (tenderness and virtue), and prioritize occupations considered appropriate for the gender group (nursing and elementary school teacher) (Smith and Parrotta, 2018; Nielsen and Madsen, 2019; Duchin et al., 2020; Smith et al., 2021).

Several studies on self-stereotyping have shown the specificity of this process for disadvantaged group members relative to privileged groups (Pickett et al., 2002; Guimond et al., 2006). According to the rejection-identification model (Branscombe et al., 1999; Latrofa et al., 2009), when individuals perceive the rejection attitude or discriminatory behavior from outgroup members, their ingroup identification might alleviate the negative impact of discrimination on mental health. Thus, discrimination from outgroups unexpectedly leads individuals to favor and protect the unity of the disadvantaged ingroup, increasing gender identity and self-stereotyping as a compensating strategy that satisfies a need to feel accepted.

Life satisfaction involves individual’s evaluation of their own life in terms of satisfaction; it is a key component of subjective well-being and is significantly correlated with individual’s mental health status (Song et al., 2018). In the current study, we intended to analyze the relationship between gender self-stereotyping and life satisfaction. Previous studies have focused mainly on group identity’s relationship to individual psychological and behavioral performance (Barker, 2009). While group identity is an important indicator of people’s willingness to internalize group stereotypes, self-stereotyping is a more direct indicator to determine whether individuals internalize gender stereotypes. Meanwhile, it has been directly demonstrated that racial self-stereotyping is positively correlated with life satisfaction (Latrofa et al., 2009; Giamo et al., 2012), but, to our knowledge, the relationship between gender self-stereotyping and life satisfaction has not been analyzed directly. Individuals with a high degree of self-stereotyping are more in line with social rules and social requirements, have a high possibility of interpersonal acceptance (Nielson et al., 2020) and interpersonal harmony (Lei et al., 2017), and experience more happiness (Latrofa et al., 2009). On the other hand, individuals with a low degree of self-stereotyping experience negative social evaluation (Seddig, 2020). Moreover, the positive relationship between self-stereotyping and life satisfaction has been verified for the Western population (Latrofa et al., 2009; Giamo et al., 2012). However, compared to those in more individualistic cultures, people in China’s collectivist culture may be more likely to voluntarily internalize gender stereotypes into self-concepts, while counter-stereotypes might face much more social pressure. Thus, it is still necessary to focus on Chinese and analyze the relationship between gender self-stereotyping and life satisfaction. Therefore, in the current study, we sought to explore the relationship between gender self-stereotyping and life satisfaction among Chinese participants and examine its mechanism. We hypothesized that gender self-stereotyping is positively associated with Chinese’ life satisfaction (H1).

### The Mediation Role of Personal Self-Esteem in the Relationship Between Gender Self-Stereotyping and Life Satisfaction

There is a lack of research exploring the mechanism by which gender self-stereotyping affects life satisfaction. The mediating role of self-esteem in the relationship between self-stereotyping and life satisfaction could be explained theoretically. Self-esteem includes many types, and different types of self-esteem reflect various sources of self-worth. Therefore, it is necessary to pay attention to the different types of self-esteem and their mediation role in the relationship between gender self-stereotyping and life satisfaction.

Self-concept includes the individual self, relational self, and collective self (Tajfel, 1982; Crocker and Luhtanen, 1990; Luhtanen and Crocker, 1992; Brewer and Gardner, 1996). Self-esteem also contains personal self-esteem (PSE), relational self-esteem (RSE), and collective self-esteem (CSE). The first, PSE, refers to the self-worth obtained from differentiated and individuated self-concept which emphasizes one’s uniqueness; it is closely associated with self-evaluation about personal attributes, such as competence, talent, and value (Tian, 2006); RSE refers to the self-worth obtained from the connections with significant others, such as family and friends (Du et al., 2012). Finally, CSE refers to individual’s evaluation and feeling of the importance of the group they belong to; group identity and intergroup interaction are important sources of CSE (Du et al., 2017).

Previous studies have found that self-stereotyping is positively correlated with CSE (Biernat et al., 1996; Oswald and Lindstedt, 2006). Individuals with high gender self-stereotyping internalize the stereotype characteristics of gender groups as their own personal characteristics, and gender identity becomes an important source of self-identity.

Gender self-stereotyping is also closely associated with PSE. According to self-categorization theory, self-stereotyping occurs on all group relevant attributes or dimensions (Turner et al., 1987). Thus, positive gender stereotypes can promote self-esteem (Oswald and Lindstedt, 2006). However, the internalization of negative group stereotypes may threaten one’s social identity and lead to a reduction in PSE (Katz et al., 2002). For the disadvantaged female group, internalization of the lesser value that women hold in society might lead to their negative personal evaluation of self, and it has been documented that girl begins to demonstrate lower PSE than do boy in adolescence (Katz et al., 2002).

Furthermore, social identity theory holds that people strive to maintain a positive social identity (Tajfel, 1982). Therefore, positive gender stereotypes are easily integrated with the self-concept and promote positive PSE. However, it is not clear how individuals can internalize negative stereotypes yet maintain positive self-evaluations. We surmised that there might be three ways that this can happen: (1) People endorse negative stereotypes as more “group-descriptive” than “self-descriptive” (Oswald and Lindstedt, 2006). (2) People ignore the negative aspects of group stereotypes. (3) People perceive negative stereotypes as unimportant and valueless for their future (e.g., low task value; for example, there are stereotypes about females being less capable or proficient than males in mathematics, but some females may think mathematics is not important for them; Song et al., 2017). Thus, we hypothesized that self-stereotyping is generally positively associated with PSE (H2a).

Moreover, PSE is a direct factor that affects people’s well-being. Many studies have documented that self-esteem plays a positive role by enhancing well-being (Quevedo and Abella, 2011; Douglass and Duffy, 2015; Yao et al., 2016). The cognitive model of depression suggests that related negative self-schemas, including feelings of worthlessness, failure, and low self-esteem, constitute a cognitive susceptibility to depression, whereas positive perceptions of self-schemas lead to high life satisfaction (Beck, 2008). Thus, we hypothesized that PSE is positively associated with life satisfaction (H2b), and PSE plays a mediating role in the relationship between gender self-stereotyping and Chinese’ life satisfaction (H2).

### The Mediation Role of Relational Self-Esteem in the Relationship Between Gender Self-Stereotyping and Life Satisfaction

There are few studies that have directly analyzed the relationship between self-stereotyping and RSE. We speculated that individuals with a high degree of gender self-stereotyping would be much more accepted by parents, family, teachers, and society than those with low gender self-stereotyping because they are more likely to meet the requirements of social norms and expectations of significant others. Alternatively, a counter-stereotypical individual might be at greater risk for interpersonal rejection. Researchers have reported that women who exhibit non-stereotypical traits and do not conform to ascribed gender roles are perceived as being more competent and having more agency than the stereotypical women (Song and Liu, 2021), yet are still more negatively evaluated at work compared to men (Brescoll and Uhlmann, 2008). Counter-stereotyped women are often perceived as interpersonally deficient (Rudman and Glick, 1999), insufficiently “nice” (Brescoll et al., 2010), and less feminine, and they are often not welcomed by their male counterparts (Song et al., 2017; Song and Liu, 2021), compared to stereotyped women. Meanwhile, counter-stereotypical men can be perceived even more negatively than counter-stereotypical women (Levy et al., 1995; Blakemore and Russ, 1997; Ed Ucational, 1999; Rudman and Mescher, 2013; Vandello et al., 2013). Some media advocate men having masculine characteristics and criticize male entertainers who use heavy makeup, wear sexy clothes, or appear as gender-confused figures. Men who have prominent “feminine traits” or engage in female-dominated fields are viewed as less competent, weak, abnormal, incapable of leadership, and less desirable as potential partners (Brescoll and Uhlmann, 2005; Wen et al., 2020). Counter-stereotyped men would face low social acceptance in present-day China, and the affirmation and acceptance of significant others are important sources of RSE. Therefore, we hypothesized that gender self-stereotyping is positively correlated with RSE (H3a).

Furthermore, a study found that there was a positive correlation between RSE and life satisfaction (Wagner, 2009). Du et al. (2014) showed that RSE was positively associated with multiple indicators of psychological well-being, and both family-related and friend-related RSEs were important to well-being. Du et al. (2017) further compared the predictive effects of RSE, PSE, and CSE on psychological well-being through four cross-sectional studies and one longitudinal study. The results showed that, when controlling for PSE, RSE was associated with greater life satisfaction, positive affect, meaning in life, happiness, and subjective vitality. Therefore, we speculated that RSE is positively associated with life satisfaction (H3b), and RSE might play a mediating role in the relationship between gender self-stereotyping and life satisfaction (H3).

Previous studies have shown a positive correlation between PSE and RSE (Du et al., 2015). Perceived relational evaluation, or the extent to which people see others as valuing them, is a particularly significant determinant of self-esteem (Norman et al., 2012). Additionally, positive evaluations from others who are considered “important” can lead improve an individual’s sense of self-worth and induce higher competence self-evaluation. In summary, we speculated that RSE was positively associated with PSE (H4), and thus, gender self-stereotyping could indirectly affect life satisfaction through RSE and PSE in a serial pattern.

### Current Study

As described above, self-stereotyping has shown the specificity of this process for disadvantaged group members relative to privileged groups (Pickett et al., 2002; Guimond et al., 2006). Therefore, the effect of self-stereotyping on females’ life satisfaction, RSE, and PSE seems much higher than for males and that the mediation model would be moderated by gender. However, other studies indirectly showed that males face higher social pressure of self-stereotyping than females. The social acceptance toward counter-stereotypical males was lower than counter-stereotypical females (Levy et al., 1995; Blakemore and Russ, 1997; Ed Ucational, 1999; Vandello et al., 2013). It has been demonstrated that boys’ gender-role violations involving physical appearance would be judged to be more serious than similar transgressions by girls (Levy et al., 1995; Blakemore and Russ, 1997). For the gender-role violation involving family/work oriented, previous research also inferred men who seek work flexibility may be particularly penalized (e.g., being demoted or downsized) and interpersonal stigmatized compared with women who seek work flexibility (Wayne and Cordeiro, 2003; Butler and Skattebo, 2004; Rudman and Mescher, 2013), as these men were seen as less masculine and rated lower on masculine prescriptive traits and higher on feminine prescriptive traits (Rudman and Mescher, 2013; Vandello et al., 2013). Thus, we could also inference that the effect of gender self-stereotyping on males’ life satisfaction, RSE, and PSE seems much higher than for females. As the contrary results in previous research, we made exploratory research and hypothesized that the serial mediation model would be significantly different in the female sample compared with that in the male sample (H5) and gender plays a moderation role in the serial mediation model.

The purpose of the current study was to analyze the direct effect of self-stereotyping on life satisfaction, the indirect effect through PSE and RSE in a serial pattern, and the moderation role of gender. To analyze the mediation role of RSE and PSE, we conducted a serial multivariable mediation (Hayes, 2013); the self-stereotyping was assumed to influence life satisfaction through three specific indirect effects: through PSE (H2), through RSE (H3), and through both RSE and PSE (H4). Moreover, to analyze the moderation role of gender in the serial mediation model (H5), we analyzed the serial mediation model in male and female samples; the difference between these two serial mediation models indicates the significance of the moderating role of gender.

## Materials and Methods

### Participants

A total of 328 college students from a university in Wuhan participated in this investigation; 317 valid questionnaires were collected, including 171 men (53.82%) and 146 women (46.18%); 127 in rural areas (40.1%) and 190 in urban areas (59.9%). The age of the participants was ranged from 17 to 26 years old (*M* = 18.36, *SD* = 1.11). For the subjective family economic situation, one participant perceived him/her as very poor (0.3%), 25 participants perceived them as a little poor (7.9%), 220 as average (69.4%), 66 as a little rich (20.8%), and five as very rich (1.6%).

### Questionnaire

#### Gender Self-Stereotyping

Gender self-stereotyping refers to the extent to which people attribute relevant group characteristics, both positive and negative, to the self (Latrofa et al., 2009). It was measured through the evaluation similarity between self and ingroup. Participants both rated self and their gender ingroup on 22 personality traits: 11 male trait words (brave, rational, competence, efficient, work-oriented, good at science and engineering, like video games, reckless, impulsive, aggressive, and careless), and 11 female trait words (gentle, emotional, warmth, kind, family oriented, good at humanities, like shopping, timid, sensitive, fragile, and emotional). We chose these traits words based on previous research (Zhang et al., 2015; Zuo, 2015). The degree of I/female or I/male have these traits was assessed on a scale from 1 = none to 7 = very much. For each participant, we could get 22 rating scores describing themselves and the 22 rating scores describing their ingroup. When calculating the self-stereotyping for female participants, the 11 male trait words were reverse-scored; when calculating the self-stereotyping for male participants, the 11 female trait words were reverse-scored.

The self-stereotyping indices for each participant were obtained by calculating correlations between self and ingroup ratings with the following equation:


$$\text{r}=\sum_{i=1}^{n}\left(X_{i}-\bar{X}\right)\,\left(Y_{i}-\bar{Y}\right)/\sqrt{\sum_{i=1}^{n}{\left(X_{i}-\bar{X}\right)}^{2}}\,\sqrt{\sum_{i=1}^{n}{\left(Y_{i}-\bar{Y}\right)}^{2}}$$

The higher the correlation between evaluation on self and ingroup, the higher the degree of gender self-stereotyping. This method of measuring self-stereotyping has been used in previous studies and has been demonstrated to be effective (Latrofa et al., 2012). In the current study, the Cronbach’s alpha coefficient of this measurement was 0.71. For the 22 items of self-evaluation, the confirmatory factor analysis showed that the model had an acceptable fit, CMIN/DF = 3.47, GFI = 0.84, NFI = 0.85, IFI = 0.89, TLI = 0.86, CFI = 0.09, RMSEA = 0.088. For the 22 items of evaluating ingroup, the confirmatory factor analysis showed that the model also had an acceptable fit, CMIN/DF = 4.50, GFI = 0.81, NFI = 0.84, IFI = 0.87, TLI = 0.82, CFI = 0.87, and RMSEA = 0.10.

#### Relational Self-Esteem

Relational self-esteem scale was used to measure the RSE (Du et al., 2012); this scale included eight items (in general, I am glad to become a member in my circle of friends); four-point rating scale was used, with 1 = strongly disagree, 4 = strongly agree. The higher score indicated high RSE. In the current study, the Cronbach’s alpha coefficient was 0.88.

#### Personal Self-Esteem

Personal self-esteem (PSE) was measured by the 10-item Rosenberg Self-Esteem Scale (Wang et al., 1999). For example, I have a positive attitude toward myself. One item (the eighth item: I wish I could have more respect for myself) was removed because of its low validity in Chinese samples (Tian, 2006; Chen et al., 2013). Items were rated on a four-point Likert scale (1 = strongly disagree, 4 = strongly agree). Higher scores indicated higher PSE. This scale’s internal consistency was good in the current sample (Cronbach’s alpha = 0.87).

#### Life Satisfaction

Life satisfaction was assessed by the Chinese version of the Student’s Life Satisfaction Scale (Huebner, 1991). Participants rated seven items (e.g., my life is going well) on a six-point Likert scale (1 = low, 6 = high). Higher scores indicated higher levels of life satisfaction. This Chinese version scale has been widely used (e.g., Hou et al., 2009). This scale’s internal consistency was good in the current sample (Cronbach’s alpha = 0.80).

### Procedure

Permission to conduct the study was granted by the Research Ethics Committee at the institution with which the first author was affiliated. College students voluntarily scanned the QR code and filled in the online questionnaire. First, the purpose and requirements of the study were explained, and then participants gave written informed consent to participate. Moreover, participants answered the questionnaire of self-stereotyping, PSE, RSE, and life satisfaction. When the study was complete, the participants were given a small reward (random red packet, from one to three CNY) to thank them for their help.

## Results

### Descriptive Statistics and Correlation Analyses

As shown in Table 1, gender self-stereotyping was positively related to PSE, RSE, and life satisfaction. Both PSE and RSE were positively related to life satisfaction. In order to avoid common methodological deviations, the Harman single factor method was used; the results showed that the first factor explained a variation of 17.84%, which was less than the 40% critical value. Therefore, the influence of common method deviation on the results of this study can be excluded (Zhou and Long, 2004).

**TABLE 1:** Means, standard deviations, and Pearson’s correlations among all variables.

	*M*	*SD*	1	2	3	4	5
1 Self-stereotyping	0.39	0.30	1				
2 PSE	27.36	4.66	0.19**	1			
3 RSE	25.39	4.18	0.17**	0.63***	1		
4 Life satisfaction	28.01	5.52	0.21**	0.57***	0.53***	1	
5 Gender	0.54	0.50	0.25**	0.03	–0.01	–0.02	1

N = 317. **p < 0.01 and ***p < 0.001. Gender was a dummy variable, with male = 1 and female = 0. M (gender) referred to the proportion of male in all participants. The correlation coefficient between gender and self-stereotyping was positive indicating that the male has a higher level of self-stereotyping compared with female participants.

### Mediation Role of Personal Self-Esteem and Relational Self-Esteem

We used Hayes’s (2013) PROCESS macro (Model 6) to examine the serial mediation model. Moreover, as previous research has generally shown that people belonging to numerical or status minorities are more likely than majority members to ascribe stereotypic characteristics to the self. Thus, gender was used as control variables in the mediation effect model (Guimond et al., 2006). In addition, we also controlled the age in the mediation effect. We used bias-corrected percentile bootstrap method with 5000 bootstrap samples to calculate the indirect effect. The indirect effect was significant at *p* = 0.05 if the 95% confidence interval didn’t include 0 (Erceghurn and Mirosevich, 2008).

As shown in Table 2, gender self-stereotyping was a significant positive predictor of RSE (β = 0.18, *p* < 0.001) and life satisfaction (β = 0.10, *p* < 0.05). The results also showed that RSE was positively associated with PSE (β = 0.62, *p* < 0.001) and life satisfaction (β = 0.27, *p* < 0.001); PSE was positively associated with life satisfaction (β = 0.38, *p* < 0.001).

**TABLE 2:** Regression results for the serial mediation model.

Dependent variables	Independent variables	*R*	*R* ^2^	*F*	β	*t*
RSE	Self-stereotyping	0.24	0.06	6.52***	0.18	3.26***
	Gender				–0.09	–1.62
	Age				–0.16	−2.87**
PSE	Self-stereotyping	0.64	0.41	53.93***	0.08	1.69
	RSE				0.62	13.96***
	Gender				0.02	0.50
	Age				0.04	0.93
Life satisfaction	Self-stereotyping	0.62	0.38	38.06***	0.10	2.18*
	RSE				0.27	4.56***
	PSE				0.38	6.49***
	Gender				–0.06	–1.23
	Age				–0.02	–0.44

* p < 0.05, ** p < 0.01, and *** p < 0.001.

The mediation analysis with bias-corrected percentile bootstrap method showed that the total indirect effects of gender self-stereotyping on life satisfaction via PSE and RSE were significant and the total indirect effect was 0.12. As shown in Table 3, the mediation role of RSE in the relationship between gender self-stereotyping and life satisfaction was significant (H2 was supported), the serial mediation role of RSE and PSE was also significant (H4 was supported), but the mediation role of PSE was not significant (H3 was not supported).

**TABLE 3:** Indirect effects and 95% CIs for the mediational model.

	Effect	Boot SE	Boot LLCI	Boot ULCI
Direct effect	0.10	0.05	0.01	0.19
Total indirect effect	0.12	0.03	0.06	0.19
Indirect effect via RSE	0.05	0.02	0.01	0.09
Indirect effect via PSE	0.03	0.02	−0.01	0.06
Serial indirect effect	0.04	0.02	0.01	0.08

### The Moderation Role of Gender

As the gender might moderate the serial mediation model, thus, we also analyzed the serial mediation model in male and female samples, respectively. For the female participants, in the model of self-stereotyping that affected the RSE, the self-stereotyping was not associated with RSE (β = -0.001, *p* = 0.99). In the model of self-stereotyping and RSE affected PSE, self-stereotyping was not significantly associated with PSE (β = 0.05, *p* = 0.43), and RSE was positively associated with PSE (β = 0.69, *p* < 0.001). In the model of self-stereotyping, RSE, and PSE affected the life satisfaction, all these three variables were positively associated with life satisfaction (β = 0.21, *p* < 0.01; β = 0.34, *p* < 0.001; β = 0.34, *p* < 0.001). Moreover, the mediation effect analysis showed that the total indirect effect of self-stereotyping on life satisfaction was not significant (*B* = 0.02, *LLCI* = -0.12, *ULCL* = 0.14), the mediation effect of RSE and PSE was also not significant (*B* = -0.0004, *LLCI* = -0.08, *ULCL* = 0.07; *B* = 0.02, *LLCI* = -0.04, *ULCL* = 0.07), and the serial mediation effect of PSE and PSE was not significant (*B* = -0.0003, *LLCI* = -0.05, *ULCL* = 0.05).

For the male participants, in the model of self-stereotyping that affected the RSE, the self-stereotyping was positively associated with RSE (β = 0.33, *p* < 0.001). In the model of self-stereotyping and RSE affected PSE, self-stereotyping was not significantly associated with PSE (β = 0.11, *p* = 0.08), and RSE was positively associated with PSE (β = 0.56, *p* < 0.001). In the model of self-stereotyping, RSE and PSE affected the life satisfaction, self-stereotyping was not associated with the life satisfaction (β = 0.01, *p* = 0.85), and RSE and PSE was positively associated life satisfaction (β = 0.23, *p* < 0.01; β = 0.42, *p* < 0.001). Moreover, the mediation effect analysis showed that the total indirect effect of self-stereotyping on life satisfaction was significant (*B* = 0.19, *LLCI* = 0.11, *ULCL* = 0.29), the mediation effect of RSE was significant (*B* = 0.07, *LLCI* = 0.01, *ULCL* = 0.15), the mediation effect of PSE was not significant (*B* = 0.04, *LLCI* = -0.003, *ULCL* = 0.10), and the serial mediation effect of RSE and PSE was significant (*B* = 0.07, *LLCI* = 0.03, *ULCL* = 0.13). In summary, the serial mediation effect of RSE and PSE in the relationship between gender self-stereotyping and life satisfaction was only significant in the male participants, the moderation role of gender was supported.

## Discussion

The current study explored the relationship between gender self-stereotyping on life satisfaction and its mechanism. The results showed that gender self-stereotyping not only directly promoted life satisfaction but also indirectly affected life satisfaction through the serial mediation role of RSE and PSE. Moreover, the serial mediation effect of RSE and PSE was only significant in the male sample. This study expanded previous research in the following aspects. First, previous research analyzed the relationship between the group identity and psychological and behavioral outcomes; we focused on the direct impact of self-stereotyping on life satisfaction. Compared with group identity, self-stereotyping is a variable that could directly reflect how strongly group stereotypes have been internalized. Second, previous studies have explored the relationship between racial self-stereotyping and life satisfaction (Latrofa et al., 2009), while our study focused on gender self-stereotyping. Third, we verified the existence of a relationship between gender self-stereotyping and life satisfaction in China. Fourth, our study systematically revealed the internal mechanism of gender self-stereotyping affected life satisfaction and analyzed the mediation role of PSE and RSE. Fifth, we also analyzed the moderation role of gender; the results indicated that the self-stereotyping process only leads to male participants having high RSE, which further leads to high PSE and high life satisfaction. Counter-gender stereotypical male might experience high social pressure and low social acceptance compared with counter-gender stereotypical female. This has been verified in a Western country long before (Levy et al., 1995; Blakemore and Russ, 1997; Ed Ucational, 1999; Rudman and Mescher, 2013; Vandello et al., 2013). We constructively and repeatedly verified this result in China samples.

### The Relationship Between Gender Self-Stereotyping and Life Satisfaction

This study found a significant positive correlation between gender self-stereotyping and life satisfaction, which is consistent with previous research (Latrofa et al., 2009). Our results also show that when individuals meet social requirements and integrate group identity into their self-concept, their life satisfaction increases. The self-stereotyping process might involve a risk of people identifying negative characteristics of their ingroup, further leading to lower self-evaluation. However, it can also create gains to ingroup identity and group belonging. Self-stereotyping individuals are more likely to be recognized and accepted by the ingroup and outgroup members, and thus they might have higher life satisfaction. Individuals who present low levels of self-stereotyping, possessing personality characteristics and behaviors in violation of gender role requirements, have a higher possibility of experiencing social exclusion and more interpersonal pressure, resulting in lower life satisfaction. Therefore, the social role deviation needs to pay a substantial social cost, and thus counter-stereotype might reduce life satisfaction.

### The Mediation Role of Personal Self-Esteem and Relational Self-Esteem

The results also showed that gender self-stereotyping can indirectly affect life satisfaction through RSE. People with a higher degree of gender self-stereotyping conform more than others to the social expectations around their gender role; they may also conform to the social expectations of “important others.” Thus, there is a high possibility that such individuals experience good interpersonal relationships and high RSE. For example, women who internalize gender roles would exhibit typical feminized characteristics and try to be good mothers and wives. They are more family oriented, care about and value their family more than work and self-actualization, and could derive self-esteem from family relationships. However, females who exhibit masculine characteristics or are successful in traditional male roles were often negatively evaluated and need to face more social exclusion. Career success might even make them less sexually attractive to men, reduce their commitment to their family; this might lead them to have a high possibility of low RSE. Therefore, there was a positive correlation between self-stereotyping and RSE.

The mediation effect of PSE on the relationship between gender self-stereotyping and life satisfaction was not significant. Gender stereotyping related to females contained positive content (females were good at reading and were gentle, careful, and kind) and negative content (females are not good at STEM; girls have low competence and lack of confidence). The internalization of positive gender stereotypes can promote PSE (Oswald and Lindstedt, 2006), while internalization of negative stereotypes can lower PSE (Katz et al., 2002). These paradoxical results may have resulted in an insignificant relationship between self-stereotyping and self-esteem.

Moreover, gender self-stereotyping can indirectly affect life satisfaction through the serial mediation of RSE and PSE. RSE refers to the self-worth gained from significant others, while PSE refers to the belief and evaluation of one’s own abilities and values. Therefore, a high RSE could enhance an individual’s sense of self-worth and promote positive self-evaluation. In general, individuals with a high degree of gender self-stereotyping conform to group norms, are in line with the expectations of important others, have higher RSE and PSE, and ultimately experience higher life satisfaction. On the contrary, a low degree of self-stereotyping means violating social expectations and not matching the requirements of social roles, which may lead to less harmonious relationships with family members and others, decrease RSE, reduce PSE, and ultimately lead to lower life satisfaction.

### The Moderation Role of Gender

We found that the serial mediation role of RSE and PSE was only significant in the male sample (not in the female sample); thus, the moderation role of gender was supported. Previous research has demonstrated that people from disadvantaged groups are more likely to feel discrimination and prejudice. The disadvantaged group identity (gender, race, etc.) is unchangeable. Identifying ingroup identity and increasing the sense of belonging becomes a protective strategy to alleviate the negative impact of discrimination from outgroup (Pickett et al., 2002; Guimond et al., 2006). Based on the review above, females have a high level of self-stereotyping, and self-stereotyping was more influential in female groups.

However, our study found completely opposite results: Gender self-stereotyping did not improve the females’ RSE, but could improve male’s RSE, and further improve PSE and life satisfaction. We speculate that this might be because: (1) The Chinese male might face higher social pressure of self-stereotyping than the female. They have to be masculine, and meanwhile, they need to prove that he is not female. A man who deviates from male roles would have a much harder time and experience lower social acceptance than counter-stereotypical female (Levy et al., 1995; Blakemore and Russ, 1997; Ed Ucational, 1999; Rudman and Mescher, 2013; Vandello et al., 2013). Although counter-stereotypical females did not meet the requirements of gender roles, they tend to be much more capable compared to stereotypical females; this change is in line with the social norm of “trying to better oneself and striving for self-realization.” However, counter-stereotypical males are considered less capable than the stereotypical male (Brescoll and Uhlmann, 2005; Vandello et al., 2013; Wen et al., 2020); they neither conform to the gender roles nor social norms of “being positive.” Therefore, counter-stereotypical males might face greater pressure of social exclusion than counter-stereotypical females, and counter-stereotypes lead to males having low RSE and PSE. (2) Women’s social roles have changed over the past quarter-century. Women have entered the labor force at higher rates than ever, have begun to take on the roles traditionally held by men, and are increasingly becoming household breadwinners (Chang et al., 2011, 2017; Lu et al., 2015). As a result, the social stereotypical expectation and requirements toward females also changed. Internalizing the traditional stereotypical female traits is not to conform to the current gender role requirement for females. Thus, the females’ gender self-stereotyping degree was lower than males, and the internalization of traditional feminine stereotypes was not associated with females’ PSE and RSE.

### Implications

Many education experts encouraged people (especially low-valued group, women) to break out of stereotypes, become whomever they want to be, and achieve self-fulfillment. It has been demonstrated that exposure to counter-stereotypic gender role models (Leicht et al., 2014), effectively emotions regulation (Johns et al., 2008), self-affirmation intervention (Wang and Yu, 2017), learn about stereotype threat (Zhang et al., 2014), identity management strategies (Guan et al., 2017) could effectively intervene the negative impact of stereotype. However, these studies ignore the reality that people are actively and voluntarily self-stereotyping. For example, some females actively show that they are not good at STEM and actively choose feminine careers. It is necessary to better answer the questions: Why do people voluntarily internalize stereotypes and what are the obstacles and pressures to deviate from their social roles.

The current study explained why people voluntarily self-stereotyping. Our results provided empirical support and expanded the self-categorization theory and social identity theory. Our results highlight that conforming to gender stereotypes can have such benefits: could increase females’ life satisfaction, could increase males’ interpersonal harmony, RSE, and PSE, and ultimately leads to increased well-being. However, counter-stereotyping males face many social and interpersonal pressures and experience low interpersonal support from significant others, leading to low RSE and PSE. Thus, male is more voluntarily self-stereotyping and afraid of deviating from the gender role than female.

Moreover, the purpose of this study is not to encourage self-stereotyping but to find a more effective method to reduce self-stereotyping and break the shackles of stereotypes for social or culturally disadvantaged members (for example, the men are disadvantaged group in verbal expression and engaging in nurse career). Based on our results, we suspected that increasing the social acceptance of counter-stereotype targets are important solutions for men to voluntarily go against gender stereotypes. Men would voluntarily break stereotypes unless society accepts counter-stereotype target. For the female, counter-gender stereotypical female has low life satisfaction compared with high self-stereotyping female. We did not find out its’ reason and mechanism in the current study; we suspected it might be family work conflict that reduces counter-gender stereotypical female’ life satisfaction, but it still needs to be further confirmed in a future study.

### Limitations

This study has limitations that need to be addressed in future studies: (1) it only focuses on RSE and PSE. Future research can also integrate CSE to explore the mediating effect of gender self-stereotyping on life satisfaction. (2) It is necessary to conduct cross-cultural research to compare the cultural differences of self-stereotyping affect life satisfaction. (3) We included both positive and negative personality traits when measuring gender self-stereotyping. This positive stereotype and presentation of double-sided information may have increased gender identity (Alt et al., 2019). Thus, our measure itself may be a priming factor that increases gender identity.

## Data Availability Statement

The raw data supporting the conclusions of this article will be made available by the authors, without undue reservation.

## Ethics Statement

The studies involving human participants were reviewed and approved by the Institute of Applied Psychology, China University of Geosciences (Wuhan). The participants provided their written informed consent to participate in this study.

## Author Contributions

JS designed the study and wrote the manuscript. JL designed the study, analyzed the data, and wrote the manuscript. YL collected the data. All authors contributed to the article and approved the submitted version.

## Conflict of Interest

The authors declare that the research was conducted in the absence of any commercial or financial relationships that could be construed as a potential conflict of interest.

## Publisher’s Note

All claims expressed in this article are solely those of the authors and do not necessarily represent those of their affiliated organizations, or those of the publisher, the editors and the reviewers. Any product that may be evaluated in this article, or claim that may be made by its manufacturer, is not guaranteed or endorsed by the publisher.
